# The Thermosensitive Injectable Celecoxib-Loaded Chitosan Hydrogel for Repairing Postoperative Intervertebral Disc Defect

**DOI:** 10.3389/fbioe.2022.876157

**Published:** 2022-06-28

**Authors:** Yukun Du, Jianyi Li, Xiaojie Tang, Yingying Liu, Guoshuai Bian, Jianzhuang Shi, Yixin Zhang, Baomeng Zhao, Hongri Zhao, Kunyan Sui, Yongming Xi

**Affiliations:** ^1^ Department of Spinal Surgery, The Affiliated Hospital of Qingdao University, Qingdao, China; ^2^ Department of Spinal Surgery, Yantai Affiliated Hospital of Binzhou Medical University, Yantai, China; ^3^ State Key Laboratory of Bio-Fibers and Eco-Textiles, College of Materials Science and Engineering, Shandong Collaborative Innovation Center of Marine Biobased Fibers and Ecological Textiles, Qingdao University, Qingdao, China; ^4^ Health Care Ward III, The Affiliated Hospital of Qingdao University, Qingdao, China; ^5^ Department of Surgery teaching and research, Binzhou Medical University, Yantai, China

**Keywords:** intervertebral disc degeneration, celecoxib, lumbar disc herniation, chitosan hydrogel, percutaneous endoscopic lumbar discectomy

## Abstract

Percutaneous endoscopic lumbar discectomy has been widely used in clinical practice for lumbar spine diseases. But the postoperative disc re-herniation and inflammation are the main reason for pain recurrence after surgery. The postoperative local defect of the intervertebral disc will lead to the instability of the spine, further aggravating the process of intervertebral disc degeneration. In this work, we successfully synthesized the thermosensitive injectable celecoxib-loaded chitosan hydrogel and investigated its material properties, repair effect, biocompatibility, and histocompatibility in *in vitro* and in vivo study. *In vitro* and in vivo, the hydrogel has low toxicity, biodegradability, and good biocompatibility. In an animal experiment, this composite hydrogel can effectively fill local tissue defects to maintain the stability of the spine and delay the process of intervertebral disc degeneration after surgery. These results indicated that this composite hydrogel will be a promising way to treat postoperative intervertebral disc disease in future clinical applications.

## Introduction

Low back pain (LBP) is one of the common conditions which everyone experiences in their lifetime (Yang;Zhang;Ma and Ding, 2020; Van Zundert and Cohen, 2021). LBP has been regarded as a significant global public health problem, which was given more attention with the development of society (Tendulkar;Chen;Ehnert;Kaps and Nüssler, 2019). Studies indicated that intervertebral disc degeneration (IDD) is the most common reason for LBP (Kos;Gradisnik and Velnar, 2019).

The intervertebral disc (IVD) is the complex tissue including the jelly-like elastic nucleus pulposus, which is surrounded by layers of collagen fibers, the annulus fibrosus (AF), together with cartilaginous and bony end-plates (Liyew, 2020; Urban and Fairbank, 2020). The NP, one of the most critical components of the IVD, provides an avascular hypoxic microenvironment to support chondrocyte-like cells in a proteoglycan and type II collagen-rich ECM, regulating disc functions and deformation (Cannata et al., 2020). The AF is the circumferential ring that withstands the tension created during IVD deformation, which is enabling the uniform distribution and transfer of compressive loads between the vertebral bodies. The cartilaginous and bony end-plates are the thin layers of hyaline cartilage placed between the IVD and the adjacent vertebral bodies, which is the main approach for nutrient supply to all IVD cells through diffusion, as well as removal of waste products. With the development of IDD, the annulus fibrosus will be broken, with the consequence of the protrusion of the nucleus pulposus (NP), which is called lumbar disc herniation (LDH) (Chen;Tseng;Sun;Chang and Chen, 2021; Deng et al., 2021). Patients with LDH usually present with symptoms such as pain, numbness, and weakness, which have a significant negative influence on their social functions. Percutaneous endoscopic lumbar discectomy (PELD) was first introduced in 1992 and has been widely used in clinical practice for lumbar spine diseases due to less invasiveness and faster recovery than traditional surgery (Aprile;Amato and de Oliveira, 2020; Hu;Zheng;Chen and Chen, 2020; Tacconi;Baldo;Merci and Serra, 2020).

The PELD can significantly release nerve compression and attenuate pain caused by the protrusion of the nucleus pulposus. But the trauma of tissue caused by the operation will induce inflammation in local tissue. Also, the defect of AF is that it can no longer keep the NP residue in place, leading to pain recurrence due to NP re-herniation. The control of postoperative pain depends on oral drugs, such as celecoxib. However, oral medication alone cannot provide effective concentrations locally, and for some patients, such as those with gastric ulcers, hypertension, and cardiovascular accident, there are unavoidable additional side effects (Kocak et al., 2019; Patel et al., 2019; Schectman, 2020). Controllable release of celecoxib could decrease potential side effects and allow less administration. Other methods, such as physical therapy strengthening and muscle training, are also considered to improve clinical symptoms after surgery, but there is no effective research and clinical effect statistics supporting this. Therefore, injecting anti-inflammatory functional materials into the defect tissue after discectomy is a feasible strategy to control the inflammation microenvironment locally (Wang et al., 2019; Borrelli and Buckley, 2020).

**FIGURE 1:**
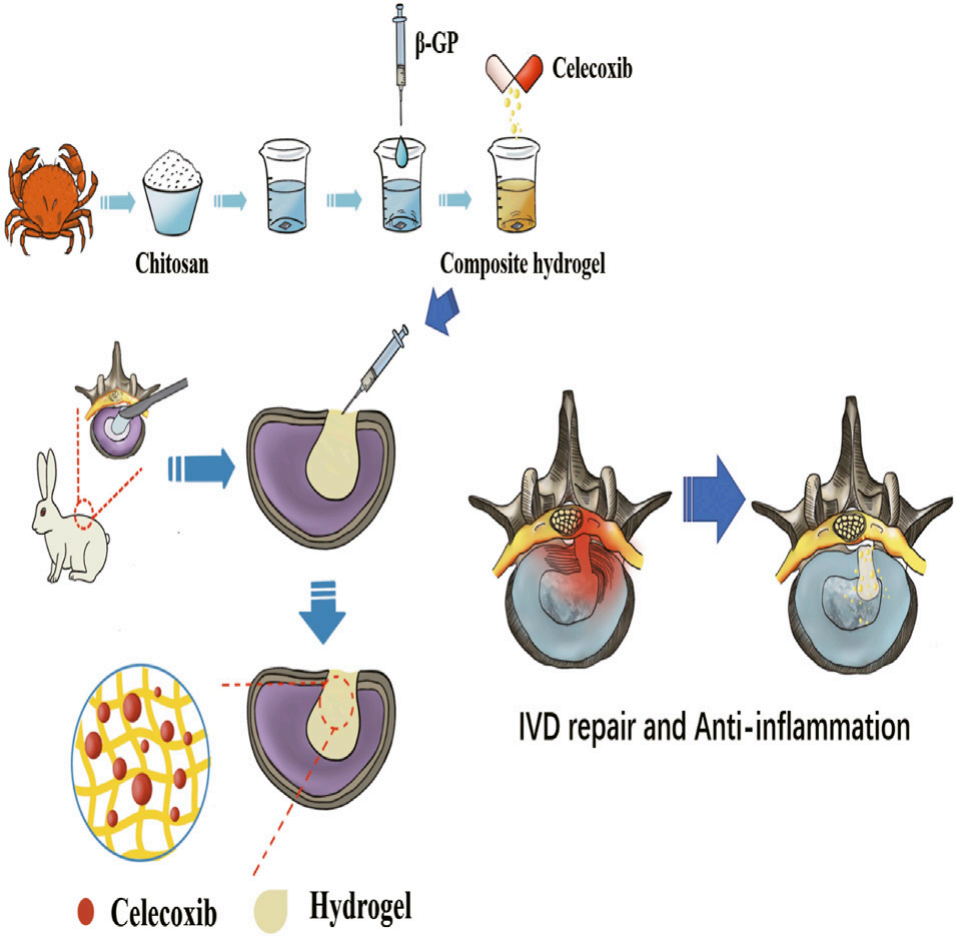
The schematic diagram of the preparation process of thermosensitive injectable celecoxib-loaded chitosan hydrogel and its effect on preventing the recurrence of lumbar disc herniation and pain after partial discectomy.

The chitosan hydrogel is a novel choice as a carrier for controllable release of anti-inflammatory drugs due to its similarity to the natural extracellular matrix and excellent biocompatibility (Alinejad et al., 2019; Zhang et al., 2021). In addition, the thermosensitive injectable chitosan-based hydrogel can be used for local treatment and is beneficial for the repair of tissue defects. Celecoxib is a new generation of non-steroidal anti-inflammatory and analgesic drugs, which is one of the commonly used anti-inflammatory and pain-relieving drugs in the orthopedic clinic. It can inhibit the production of prostaglandins by selectively inhibiting cyclooxygenase-2 (COX-2) to achieve anti-inflammatory and analgesic effects. The hydrogel delivery system could increase the release of celecoxib and further improve bioavailability, avoid burst drug release, and reduce toxicity and side effects. In this study, we designed a thermosensitive injectable chitosan hydrogel-based celecoxib (TICHC) delivery system with injectability and fixation properties to prevent NP re-protrusion and degeneration after discectomy. After injection, the thermosensitive composite chitosan-based hydrogel could repair the defect and provide the necessary mechanical support after being crosslinked, alleviating the degeneration process aggravated by mechanical changes. The locally released celecoxib could inhibit the postoperative inflammatory response of residual tissue and effectively delay the development of degeneration (Figure 1). Finally, the thermosensitive injectable chitosan-based hydrogel has been widely applied and intensively studied, and its safety and reliability have been widely verified, thus supporting its clinical application in the future.

## Methods and Materials

### Materials

Celebrex (CAS: 169590-42-5), chitosan (degree of deacetylation: 95%; molecular weight: 20 w; CAS: 9012-76-4), and β-glycerophosphate (β-GP) (CAS: 154804-51-0) were all purchased from Sigma-Aldrich (St. Louis, United States).

### Synthesis of TICHC

The 2, 3, and 5 g chitosan powder were, respectively, weighed and dissolved with 0.1 mol dilute hydrochloric acid. A total of 5.6 g of β-glycerophosphate sodium (β-GP) powder was weighed and dissolved in 10 ml ultrapure water with the assistance of magnetic stirring. A 56% β-GP solution was obtained and sterilized by using a filter membrane (22 μm) and stored in the refrigerator at 4°C for further experiment. A total of 1 g of Celecoxib powder was weighed and dissolved in 50 ml ultrapure water with the assistance of magnetic stirring. 1 ml of 56% β-GP solution was, respectively, added to 9 ml of 2%, 3%, and 5% chitosan solutions with slow stirring. According to the previous relevant literature, a total of 1–1.5 ml of 2% celecoxib solution was added to 9 ml of 2% chitosan solution with constant stirring (Choi et al., 2020).

### Characterization of TICHC

After freeze-drying, the chitosan hydrogel samples with different concentrations were fixed on the specimen platform. The samples were covered with gold produced by the sputter coater, and its morphology was observed by using a scanning electron microscope (SEM). Different parts of the hydrogels were randomly selected for further analyses of celecoxib and the hydrogel. Therefore, the hydrogels were analyzed by using an energy dispersive spectrometer (EDS) to observe the distribution of celecoxib in chitosan hydrogels. After coating gold on the surface of the sample, samples were placed into the sample chamber of the scanning electron microscope, and its morphology was observed with 15 kV accelerating voltage. The qualitative and semi-quantitative analyses of samples were conducted by using an X-ray energy spectrum analyzer.

### The Temperature Sensitivity Study of TICHC

According to the former study, a total of 3 ml TICHC was placed into sample tubes. Then, the tubes were placed in 37°C incubators to analyze temperature-sensitive characteristics of the hydrogel using the tube inversion method.

### The Injectability Study of TICHC

Samples of 5 ml liquid 2% TICHC were, respectively, extracted by using a 10-ml syringe. The 10-ml syringe was used to inject hydrogel with the appropriate force to simulate the injection process *in vivo*.

### Cell Viability

In order to evaluate the cytocompatibility of TICHC samples, the cytotoxicity was analyzed by using the cell counting kit (CCK; Sigma-Aldrich Co., Ltd., United States) and the Hoechst staining experiment (Sigma-Aldrich Co., Ltd., United States), respectively. The different extraction concentrations of hydrogel samples were divided into three groups including the control group, 50% extraction group, and 25% extraction group. The cultured L929 cells were dissociated to obtain a single cell after adding trypsin. After 1, 3, and 5 days of culture, the fresh medium and CCK reagent were added and incubated for 30 min at 37°C after the removal of the old medium. Then, the measurement of absorbance was conducted at 450 nm. After adding the hydrogel solution for 5 days, 10 ug/ml Hoechst 33342 staining solution was added to the plate at 37°C for 15 min. The cell cultured glass was fixed with 4% paraformaldehyde for 15 min and then washed with PBS solution three times to be observed under a fluorescence microscope (Leica DMI4000B, Germany).

### The Biocompatibility and Degradability Analysis

Eighteen female New Zealand rabbits (6 months old, approximately 2.0–2.5 kg) were provided from the animal center of Binzhou Medical University (Yantai, China) and were used to analyze the biocompatibility and degradability of hydrogel *in vivo*. Eighteen rabbits were divided into 3 groups, 6 in each group. They were the control group, the hydrogel group, and the drug-loaded hydrogel group, respectively. About 3 ml of normal saline, chitosan hydrogel, or drug-loaded hydrogel was injected subcutaneously in the front of the thigh of each New Zealand white rabbit, depending on its group. After injection, 2 rabbits in each group were selected and the skin of the right thigh was cut at 2 weeks, 1 month, and 2 months after injection. The histocompatibility and degradation of the hydrogel samples *in vivo* were observed.

### Animal Surgery

This study was approved by the Affiliated Hospital of Qingdao University Ethics Committee. The animal experiment and feed were in accordance with the guidelines of the animal center of the Binzhou Medical University, Yantai, Shandong. The fifteen New Zealand rabbits (5–6 months, 2.0–2.5 kg) in the in vivo study were obtained from the animal center of Binzhou Medical University. They were divided into three groups based on the different times including 2 weeks, 1 month, and 2 months. Five rabbits were selected to conduct the X-ray and MRI at different times. All rabbits were housed in single cages under controlled conditions (17–23°С, and 30–70% air humidity with appreciative air circulations) and provided with adequate feed and water. All rabbits fasted for 12 h and were forbidden water for 6 h before anesthesia. Each rabbit was fixed into the anesthesia box to be anesthetized by urethane through the marginal ear vein and the penicillin was injected before the operation. The rabbit was placed on the small operation table in the left lateral position. After the removal of the fur from the dorsal surface, the anterolateral intervertebral disc 2–6 were, respectively, exposed through an anterolateral approach by blunt dissection of muscles. The IDD model of rabbits was obtained by using a 20G needle puncture at L3/4, L4/5, and L5/6, respectively. Each disc was punctured five times by using a needle and continuously aspirated with a 10-ml syringe for 30 s. The annulus fibrosus was punctured to establish the local defect, and the nucleus pulposus was partially sucked by the needle. In order to avoid individual differences, the different lumbar segment of the rabbit was conducted with different interventions on the same rabbit. A total of four groups were established including a control group (L2/3), a degeneration group (L3/4), a chitosan hydrogel treatment group (L4/5), and a chitosan hydrogel–loaded with celecoxib treatment group (L5/6). The 1-ml injection with an 18G needle was used to inject the hydrogel into different intervertebral discs. After the injection of hydrogel, the incision was sutured and the rabbits were placed on the operation table at a constant temperature to wait for recovery. The rabbits were injected with penicillin for 3 consecutive days after surgery.

### The X-Ray and MRI Evaluation

The X-ray can show the height changes of each segment to indirectly prove the instability. After 2 weeks, 1 month, and 2 months following the establishment of the IDD model, five rabbits were randomly selected for X-ray and magnetic resonance imaging (MRI) assessments at each time point. The X-ray imaging was performed by an X-ray system (Siemens, German), and the MRI tests were conducted using a 3.0-T MRI system (Siemens, German).

The X-ray and MRI image analysis and measurements were analyzed by two independent radiologists. The Bradner disc index (BDI) was used to evaluate the intervertebral height changes. The modified Thompson classification was used to evaluate the disc degeneration changes by using the T2-weighted MRI images with grades I to IV.

### Histological Analysis

The air embolism after anesthesia was used to euthanize the rabbits after 2 months. The spine samples were fixed by using formalin (10%) for 1–3 days, and the different intervertebral discs were, respectively, cut off from the spine. We used EDTA (10%) to decalcify the surrounding bone of intervertebral discs for 1–2 months. We selected the coronal median section of the intervertebral disc for the histological examination. After obtaining the wax-embedded intervertebral disc samples, we, respectively, stained the samples with H&E. The histological classification was conducted based on the observation by using light microscopy.

### Statistical Analysis

The data are shown as the mean ± standard deviation (SD). All the experiments were conducted at least three times independently. The data of results were analyzed by SPSS 23.0 statistical software. The statistical significance of the differences between the groups was calculated by using the one-way ANOVA and Tukey’s post-hoc test or nonparametric test. *p* < 0.05 is considered to be statistically significant.

## Results

### Preparation and Characterization of TICHC

**FIGURE 2:**
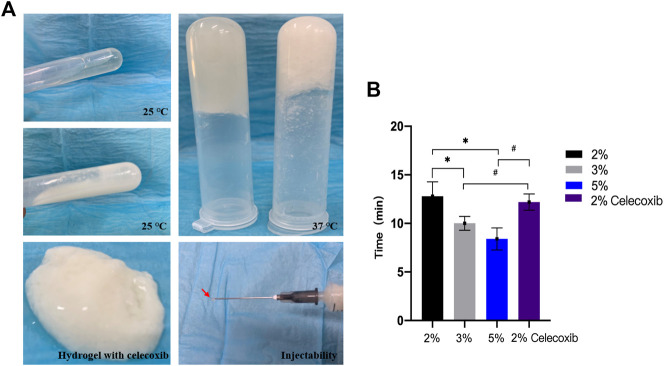
**(A)** Photographs of *in situ* gel formation of 2 wt% hydrogels (upper left) and hydrogel with celecoxib (middle left) solution with temperature increased from 25°C to 37°С (upper right), gel stability (below left), and injectability (below right). **(B)** The thermosensitive sol-gel conversion time of hydrogel solutions with different concentrations. (*,# = *p* < 0.05).

**FIGURE 3:**
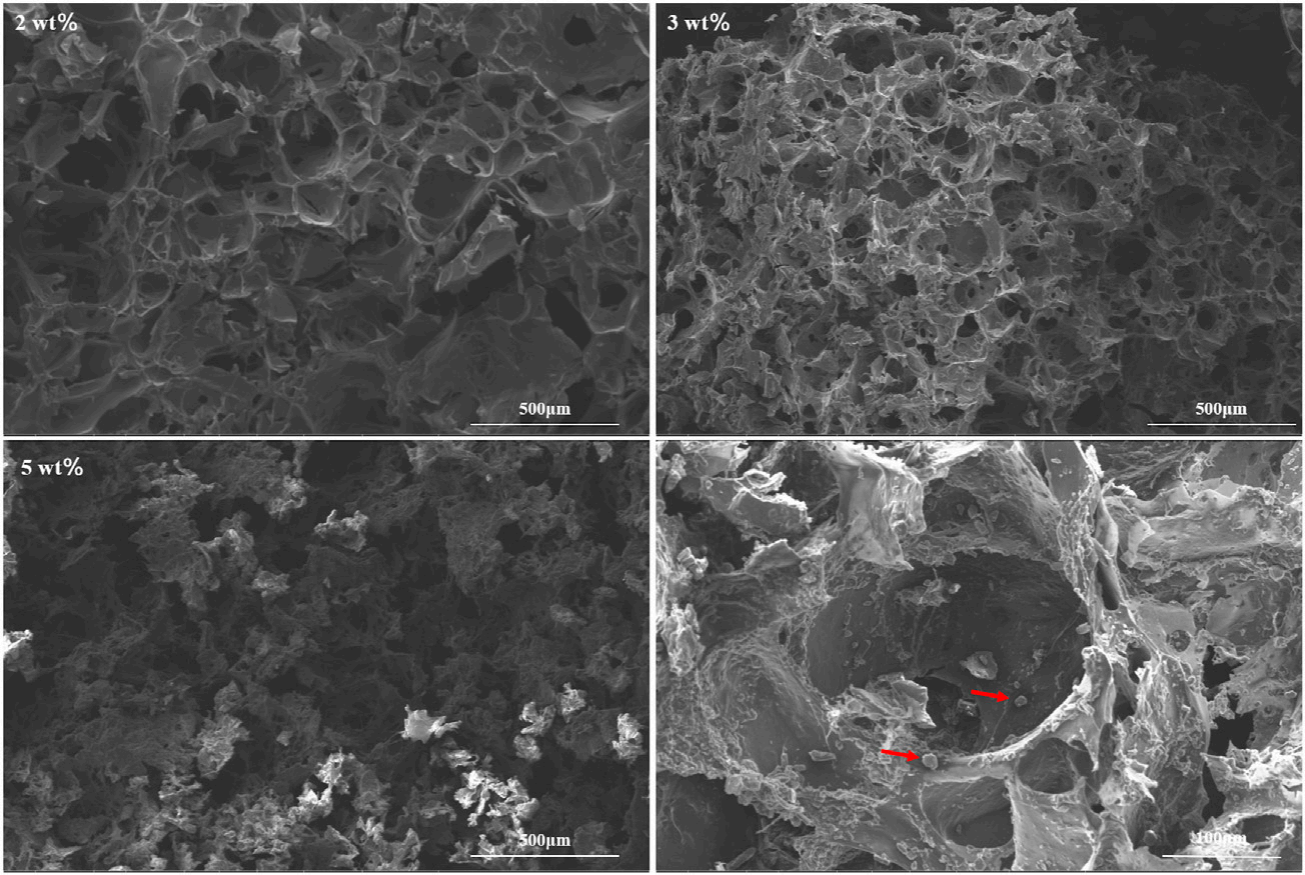
Morphologies of hydrogels with different concentrations of 2, 3, and 5 wt% and celecoxib are observed in the porous hydrogel structure (red arrow).

**FIGURE 4:**
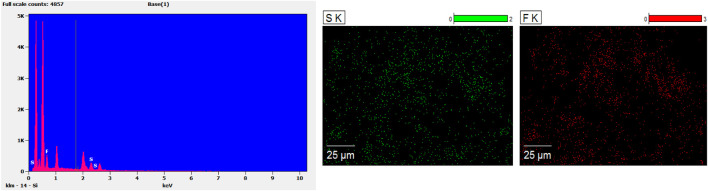
The EDS results showed the F (red) and S (green) were evenly distributed in the hydrogel, and indirectly indicated that the hydrogel had successfully encapsulated celecoxib.

The different concentrations of chitosan hydrogel in this study were the liquid state with different viscosity at room temperature. The appearance of chitosan hydrogel loaded with celecoxib was a milk-white liquid at room temperature. For this reason, it can be injected by using a different-sized injector. As shown in Figures 2A,B, the thermosensitive chitosan liquid hydrogels with/without celecoxib were able to turn solid at 37°C in 8–15 min. Hence, it was suitable for the repair of intervertebral disc defects through injection in a narrow surgical space. The thermosensitive chitosan liquid hydrogel was able to convert to a solid state at a local defect under the normal body temperature in a short time. As shown in Figure 3, the different concentrations of hydrogel showed the porous structure with different densities (higher the concentration, higher the density) by scanning electron microscopy. After comparing the different concentrations of chitosan hydrogel, the 2% hydrogel was more suitable for injection and better porous density. As shown in Figure 4, the F (red) and S (green) elements were uniformly distributed within the chitosan hydrogel by EDS, and it was indirect proof that the celecoxib was successfully loaded in the chitosan hydrogel.

In order to evaluate its liquid–solid transformation ability, we used the tube inversion method and different time measurement methods to analyze the liquid–solid conversion time of different hydrogels. As shown in Figure 2B, the results showed that the liquid–solid conversion time of 2% chitosan hydrogel with/without celecoxib was, respectively, 12.20 ± 0.84 min and 12.80 ± 1.48 min. So, the celecoxib was not able to significantly affect the liquid–solid conversion time of hydrogel. The shortest liquid–solid conversion time was 8.40 ± 1.14 min (5% chitosan hydrogel). The results indicated that the higher concentration chitosan hydrogel had a shorter liquid–solid conversion time. (*p* < 0.05).

### *In vitro* Toxicity Evaluation

**FIGURE 5:**
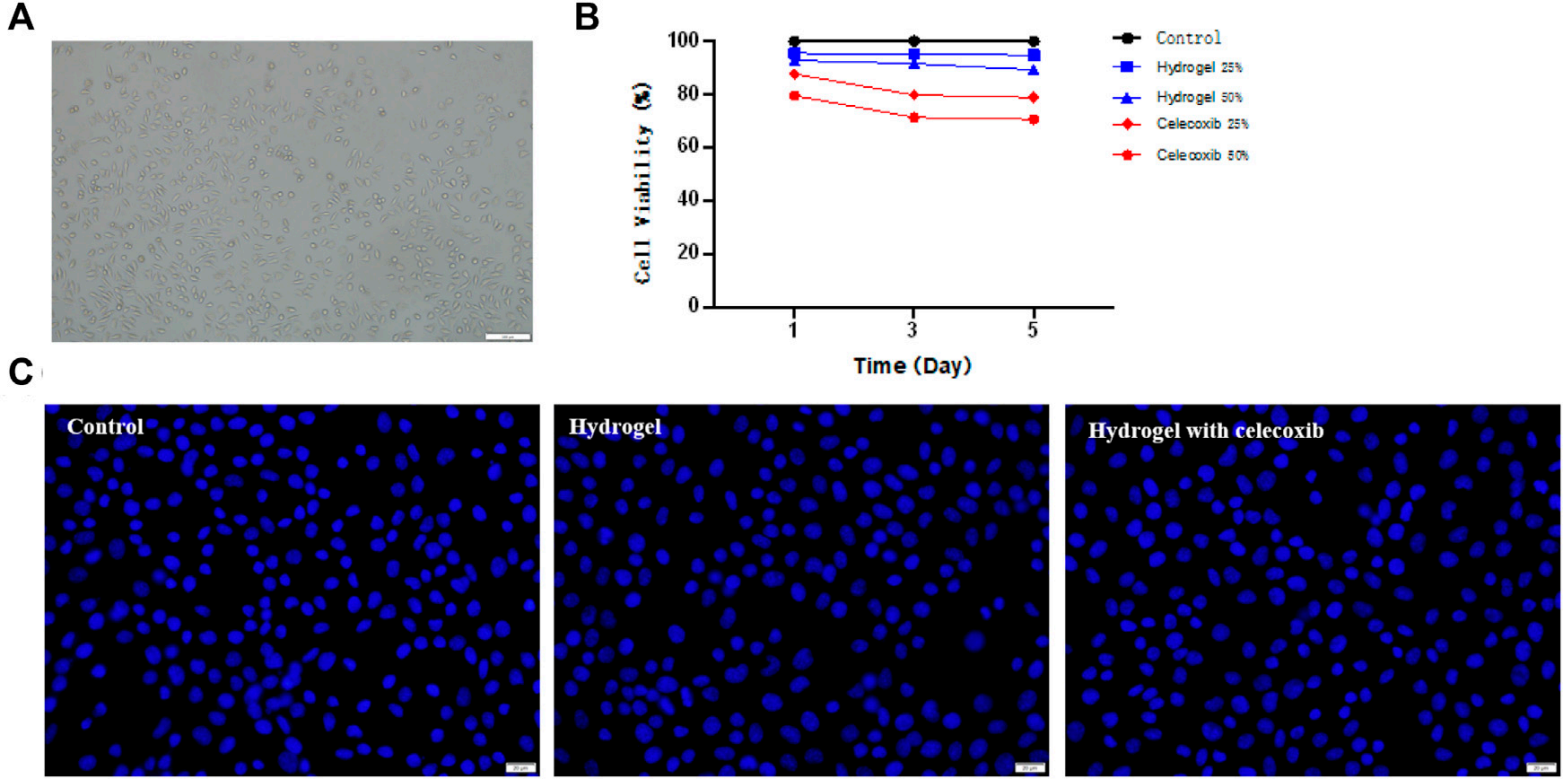
**(A)** The culture and proliferation of L929 cells. **(B)** The CCK-8 test of hydrogels. **(C)** The Hoechst staining experiment of cells cultured on the different groups.

The cytotoxicity of hydrogel was analyzed by using L929 cells. As shown in Figures 5A,B, the chitosan hydrogel with/without celecoxib had no significant effect on the proliferation of L929 cells than the control group at 1, 3, and 5 days of incubation. Moreover, the high concentration extracts of chitosan hydrogel loaded with celecoxib slightly inhibited the proliferation of L929 cells, displaying 79.56 ± 3.55%, 71.35 ± 1.39%, and 70.56 ± 6.10% cell viability for the hydrogel solution with the prolongation of culture time. Overall, the hydrogel with/without celecoxib solutions showed low cellular toxicity.

A live/dead staining assay of L929 cells was conducted using Hoechst 33342 to further confirm the cellular toxicity of hydrogels. Hoechst 33342 is a blue fluorescent dye with low toxicity, which is able to penetrate the cell membrane of the cell. The fluorescence microscopy was used to observe the proliferation of L929 cells after dyeing. As shown in Figure 5C, the results showed that the cell numbers of the three samples had no significant difference. Compared with the control group, there was no significant reduction of cell numbers in the hydrogel with/without celecoxib simples. The results indicated that the hydrogel loaded with celecoxib has satisfying biocompatibility and is a suitable injectable biomaterial for biomedical fields.

### *In vivo* Toxicity Evaluation

**FIGURE 6:**
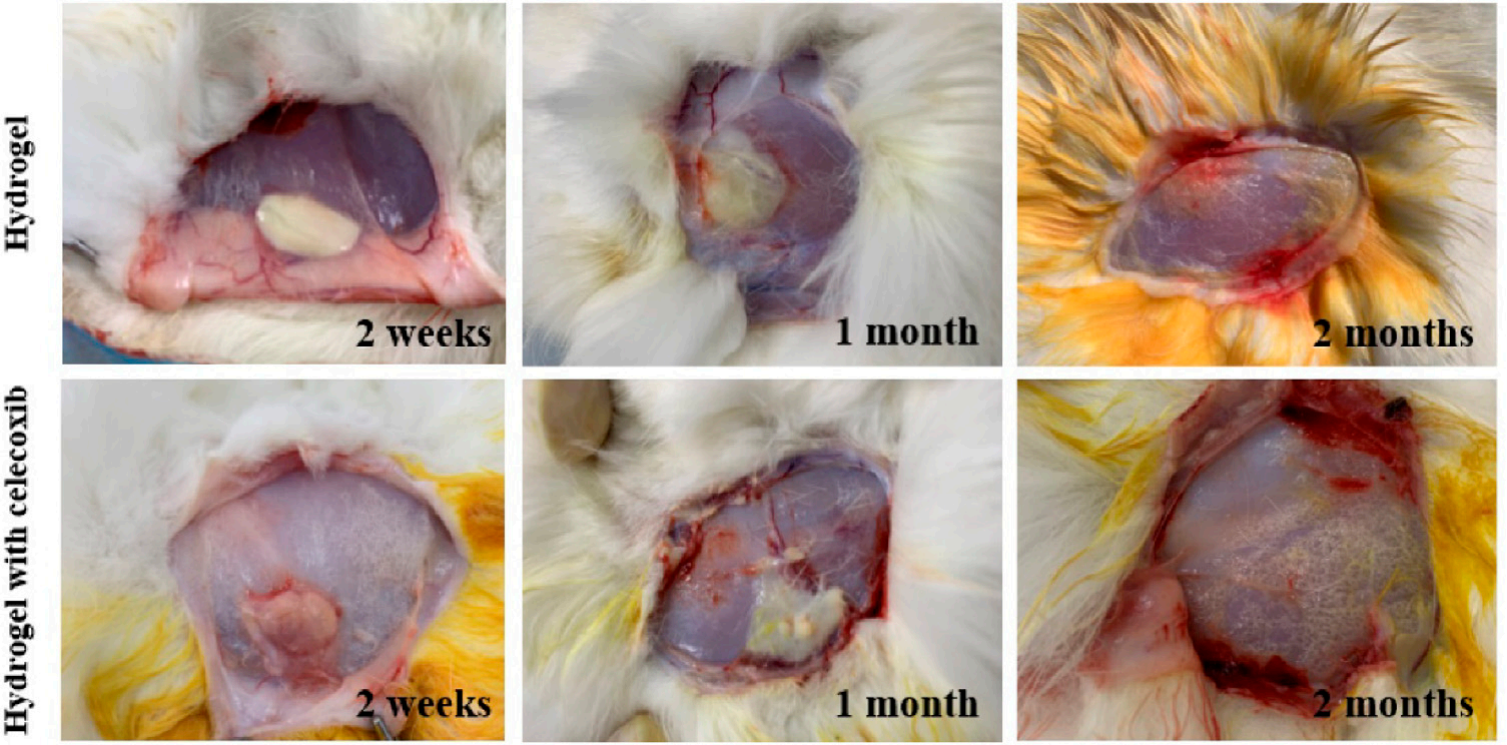
The degradation and histocompatibility analysis of hydrogel implanted subcutaneously at time of 2 weeks, 1 month, and 2 months after injection.

There was no significant infection in 18 rabbits after local hydrogel injection. In 2 weeks, there was no normal saline residue under the skin of the rabbits in the control group. No significant infection was found in the subcutaneous fascia and surrounding muscles. So, it was not necessary for further observation of the rest rabbits in the control group. As shown in Figure 6, there was obvious hydrogel residue under the skin of the rabbits of the other two groups for 2 weeks. Moreover, there was no sign of infection with the obvious distinction between hydrogel residue and surrounding tissue. With the extension of observation time, the hydrogel residues were gradually degraded under the skin, and there was no obvious hydrogel residue under the skin in 2 months. The results indicated that the injectable thermosensitive celecoxib-loaded chitosan hydrogel was able to be used as non-toxic material with good biocompatibility and histocompatibility for biomedical application.

### *In vivo* Study by X-Ray and MRI

In a total of two New Zealand white rabbits, death occurred in the process of anesthesia and post-operation. There was no obvious sign of infection, and all rabbits were in good condition after surgery. The postoperative X-ray and MRI showed the success of the establishment of the intervertebral disc degeneration model by needle puncture. Therefore, the composite hydrogel showed good sealing properties of the AF defect by injecting the exogenous hydrogel.

**FIGURE 7:**
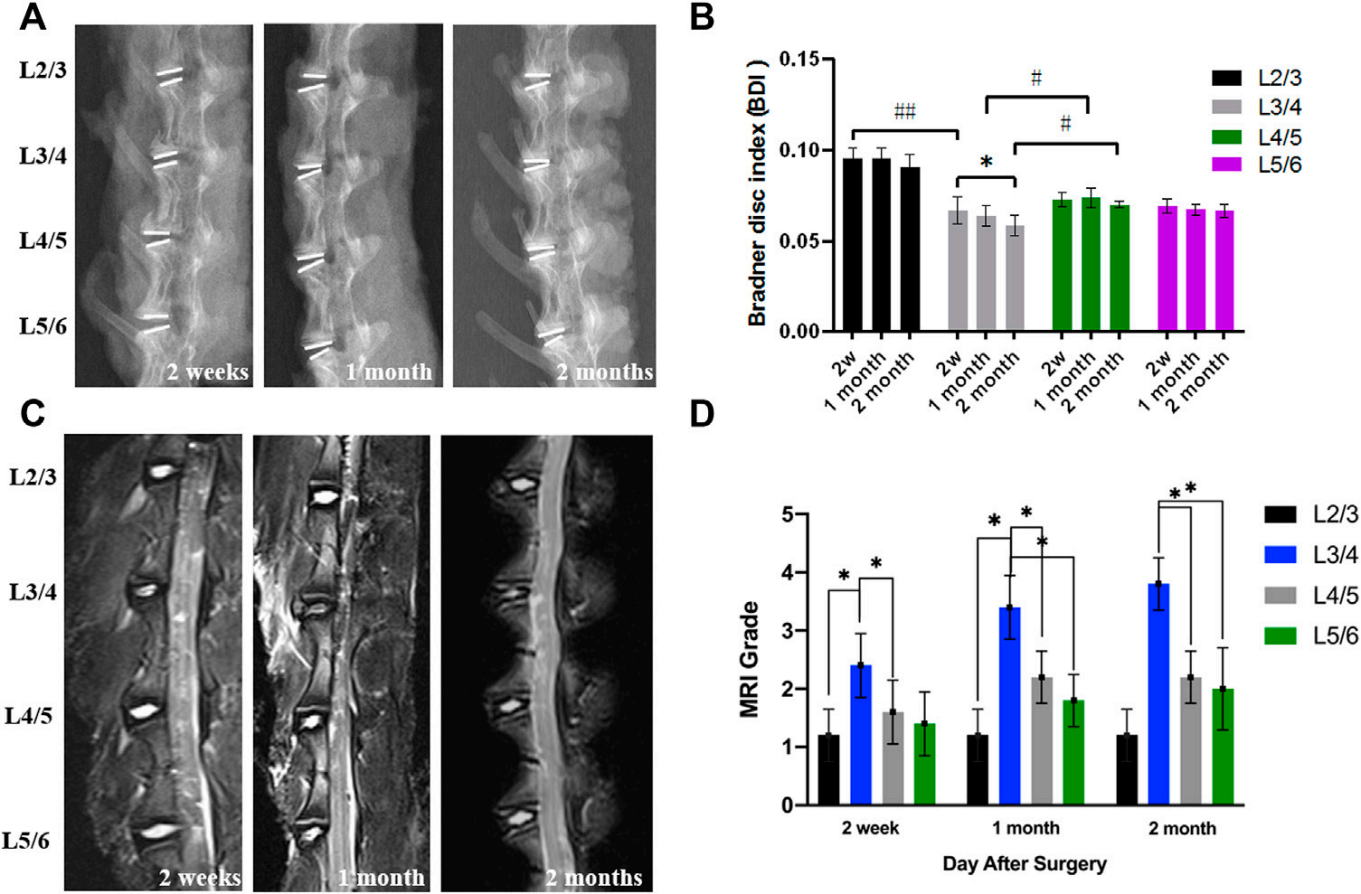
**(A)** The representative X-ray images. **(B)** The BDI changes in different groups after surgery. **(C)** The representative MRI shows the intervertebral disc signal intensity of different groups. **(D)** The MRI grade changes of different groups after the operation (*,# = *p* < 0.05).

Postoperative X-rays can visually display the intervertebral disc height. The postoperative intervertebral disc height changes were judged using the Bradner disc index (BDI). In addition, the intervertebral disc degeneration can be evaluated by the observation of sagittal MRI T2-weighted image. At 2 weeks, 1 month, and 2 months after the initial surgery, the X-ray imaging showed that a relatively normal BDI was maintained in the hydrogel treatment groups than in the degeneration group (Figures 7A,B) (*p* < 0.05). Moreover, the degeneration group had a weaker MRI signal than the hydrogel treatment groups after the operation (Figures 7C,D). With the extension of time, the difference became more obvious. However, the intervertebral height and the degeneration grade of IVD still gradually decreased in hydrogel groups than in the degeneration group.

### The Histological Analysis of Intervertebral Discs

**FIGURE 8:**
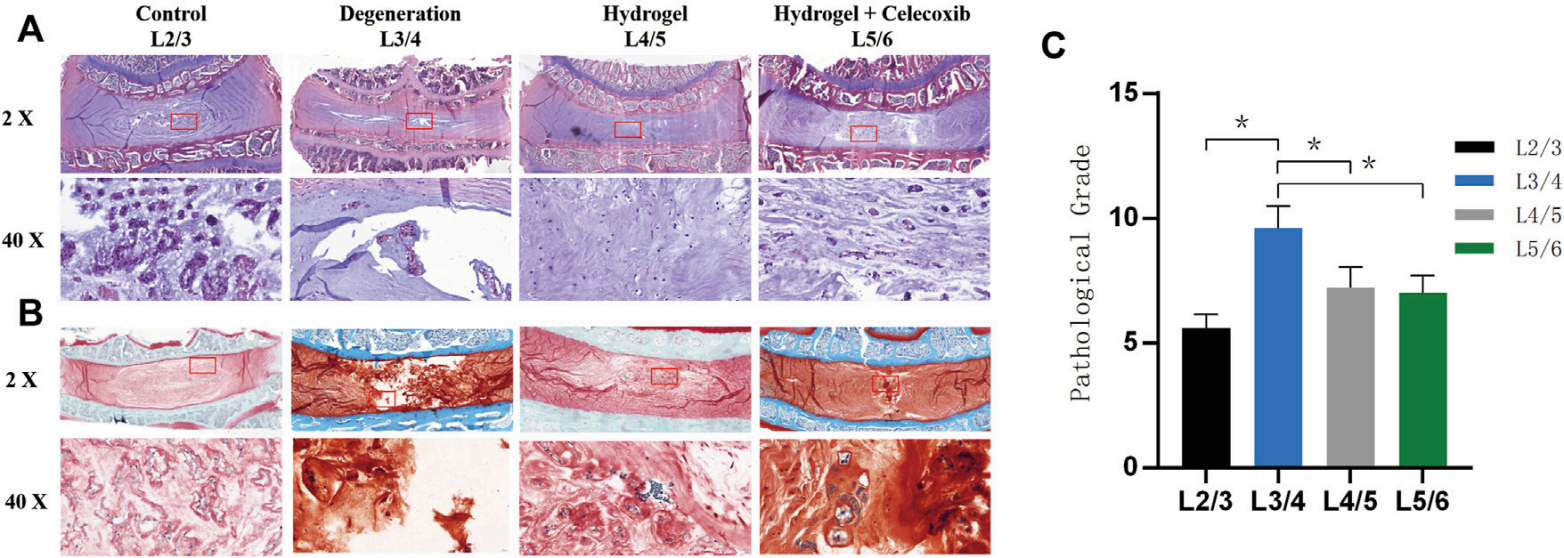
Morphological staining *in vivo*. **(A)** The representative paraffin sections with H&E. **(B)** The representative paraffin sections with H&E. **(C)** The histological grades of different groups after the operation (*,# = *p* < 0.05).

The H&E staining (Figure 8A) and safranin O-fast green staining (Figure 8B) were able to show the chromatin in the cell nucleus, cytoplasmic ribosome, cytoplasm, and the extracellular matrix. Normal nucleus pulposus tissue after staining can clearly show the NP cells and onion-shaped AF. As shown in Figure 8, the staining showed an obvious decrease in NP cells and height of IVD with a local defect in the degeneration group. However, there still was NP cell residue in the center of IVD without an obvious local defect in the hydrogel loaded with the celecoxib group. The histological grade was 9.6 ± 0.9 in the degeneration group and 7.0 ± 1.0 in the hydrogel loaded with the celecoxib group (Figure 8C). In the hydrogel loaded with the celecoxib group, there was no significant difference in histological grade compared with the hydrogel group, but it was worse than that in the control group. In addition, the number of NP cells can be observed by staining. Compared with the degeneration group, there were more NP cells residual in hydrogel groups which is beneficial for delaying the progress of IVD.

## Discussion

According to some studies, the recurrence of lumbar disc herniation is one of the main reasons for postoperative leg pain after lumbar discectomy (Hao et al., 2020; Paulsen;Rasmussen;Carreon and Andersen, 2020). At present, most scholars think the local defect of the annulus fibrosus is one of the pathogeny element of the recurrent lumbar disc herniation and reoperation. Because of the postoperative annulus fibrosus defect, the residual nucleus pulposus has a high risk of disc prolapse after surgery, especially for young patients with strong activities. Some studies showed that the incidence of recurrent lumbar disc herniation could reach nearly 62% after surgery (Huang;Han;Liu;Yu and Yu, 2016; Yaman;Kazancı;Yaman;Baş and Ayberk, 2017; Shin;Cho;Kim and Park, 2019). In addition, the intervertebral disc degeneration will be accelerated due to the damage caused by PELD, leading to the local spinal instability. Thus, the effective measures to prevent the re-protrusion of the intervertebral disc are the key issues for surgeons after PELD.

In addition, inflammation is also an important aspect that cannot be neglected in postoperative leg pain. Studies showed that the inflammatory response will be formed by the accumulation of neutrophils and lymphocytes (Djuric et al., 2019; Peng et al., 2019). The molecular mediators of inflammation, such as IL-1β, IL-6, and TNF-α, will be released to cause the inflammation (Huang et al., 2018;Ford;Kaddour;Gonzales;Page and Hahne, 2020). In fact, studies also showed that the degeneration grade of IVD was aggravated by the accumulation of inflammatory cytokines. TNF-α and IL-1β are the important inflammatory factors for intervertebral disc degeneration, which can induce intervertebral disc degeneration by reducing the anabolism of extracellular matrix proteins (Chen;Hodges;James and Diwan, 2021; Kim;Hong;Lee;Jeon and Ha, 2021). TNF-α is able to inhibit the production of extracellular matrix and increase the expression of MMP-3, MMP-9, and MMP-13. The extracellular matrix synthesis could decrease because of affected mitochondrial function and protein synthesis caused by inflammatory mediators (Lambrechts et al., 2021). Moreover, the expression of MMPs and ADAMTSs could also be affected by inflammatory mediators, leading to the degradation of collagen-2 and aggrecan. Spinal instability can be caused by the decreased extracellular matrix and reduced moisture content. Due to the important pathological role of inflammation after discectomy, it is very important to control postoperative inflammation.

Celecoxib is a selective COX-2 inhibitor, with the effect of anti-inflammatory and analgesic (Choi et al., 2020). Due to the characteristics of its chemical structure, it can be combined with COX-2 to inhibit COX-2 in the conversion of arachidonic acid to prostaglandins. It has a good anti-inflammatory analgesic with the protection of gastric mucosa. However, studies showed that systemic medication can increase the risk of serious cardiovascular thrombotic events including myocardial infarction and stroke for patients with pre-existing cardiovascular disease (Zhou et al., 2020). Some studies have already shown that the local management using celecoxib not only had an anti-inflammatory effect but also avoided the systemic adverse effects (Salgado;Guénée;Černý;Allémann and Jordan, 2020).

Considering the narrow operation space, we designed and developed the injectable thermosensitive composite chitosan-based hydrogel as the drug delivery system to attenuate local inflammation and improve degeneration in order to meet the clinical requirement. The composite hydrogel also can sustain mechanical stability by repairing the local defect after surgery. Unlike other light-sensitive materials, our thermosensitive chitosan-based hydrogel can crosslink at body temperature in a short time. We can also use electrocoagulation to reduce the crosslink time in operation.

Chitosan is widely used in biomedicine and preparation because of its biodegradability, low toxicity, and good biocompatibility (Gullbrand et al., 2017; Li et al., 2018). The degradation products of chitosan can be absorbed without accumulation or immunogenicity in the body (George;Tandon and Kandasubramanian, 2020; Qu and Luo, 2020). In recent years, studies on the role of chitosan in cartilage repair are also gradually being carried out (Rusu et al., 2019; George;Tandon and Kandasubramanian, 2020). NP cells are the main components of NP, belonging to the chondroid cells, which can secrete aggrecan and type-II collagen to synthesize extracellular matrix and then maintain the stability of NP (Liu et al., 2020). Theoretically, the gel made of chitosan is also suitable for the nucleus pulposus scaffold of the IVD nucleus pulposus cells. Roughley et al. inoculated and cultured NP cells on the prepared chitosan hydrogel (Roughley et al., 2006). They found that chitosan hydrogel has no obvious inhibitory effect on the proliferation of nucleus pulposus cells, maintaining its phenotype and promoting the growth of the extracellular matrix. In this study, these advantages of chitosan allowed NP cells to normally proliferate and had no obvious adverse effects on cells. The in vivo experiment results also indicated that the chitosan-based hydrogel had effects on the delay of degeneration of IVD to some extent. In addition, the crosslinked chitosan hydrogel is able to maintain a specific local concentration of celecoxib. The porous structure of chitosan hydrogel can provide space to contain and protect celecoxib to improve its bioavailability. In addition, the slowed degradation of the hydrogel further prolonged the drug release time.

All in all, postoperative leg pain can be caused by numerous reasons. Because of the complex structure and function of the intervertebral disc, the in vitro and animal experiments hardly simulate the actual situation encountered in clinical practice. Indeed, biomechanical tests can well explain the problem of preventing re-herniation, and biomechanical tests will continue to be added in the future. We will try large mammal animal models (such as monkeys and sheep) to better evaluate the application potential of more bioremediation materials. In the future, it is also necessary to explore the research of biological materials to repair intervertebral discs at the genetic level.

## Conclusion

In this experiment, we successfully synthesized a chitosan-based hydrogel loaded with celecoxib with thermo-sensitivity, injectability, and anti-inflammatory characteristics. The composite chitosan-based hydrogel, with the characteristics of biodegradability, low toxicity, and good biocompatibility, is able to repair the defect of IVD to prevent postoperative recurrence of disc herniation, to further maintain the stability of the spine, and to delay the IDD to some extent.

## Data Availability

The raw data supporting the conclusion of this article will be made available by the authors, without undue reservation.
